# Study protocol: behaviour change intervention to promote healthy diet and physical activity in overweight/obese adults with diabetes attending health care facilities in Muscat: a cluster rendomised control trial

**DOI:** 10.1186/s12889-021-11549-3

**Published:** 2021-08-10

**Authors:** Thamra Al Ghafri, Huda Anwar, Eiman Al Hinai, Thuraya Al Harthi, Fathiya Al Jufaili, Reyadh Al Siyabi, Shamsa Al Harthi, Said Al Hasani, Mohammed Al Harthi, Saud Al Harthi

**Affiliations:** Directorate of health services, Muscat governorate, Oman Ministry of Health, PO Box 2723, Postal Code 112 Muscat, Oman

**Keywords:** Phone counselling, Phone application, Physical activity, Healthy diet, Type 2 diabetes, Primary care, Oman

## Abstract

**Background:**

Healthy behavior is an essential component in type 2 diabetes (T2D) management. Promoting healthy lifestyle is one of the priorities of primary health care in Oman. This study aims to evaluate the effectiveness of a multi-component intervention in promoting physical activity (PA) and healthy diet and its implications on body mass index and glycemic control in adults with diabetes attending primary care.

**Methods:**

A one year 1:1 cluster randomized controlled trial will be utilized to compare the use of phone consultations, a multi component interactive phone application and pedometers with the usual diabetes care on promoting PA and healthy diet. Participants will be screened for inactivity and should be T2D, aged18–65 years, and overweight or obese. Eight primary centers will be randomly selected in each arm (*n* = 375). The primary outcome is the between arms differences in PA and diet scores, BMI and HbA1c over 12 months from baseline. Additionally, secondary outcomes will include cardiovascular outcomes (BP, and lipids).

The trial has received ethical approval from the Omani Research and Ethical Review and Approval Committee. All eligible participants will be invited to their respected health centers to provide informed consent.

**Discussion:**

This study will contribute to the integration of healthy lifestyle approach using artificial intelligence to primary diabetes care. Results from this study will be disseminated through workshops, policy briefs, and peer-reviewed publications, local and international conferences.

**Trial registration:**

Trial registration number ISRCTN71889430. Date applied: 28/11/2020. Date assigned: 01/12/2020.

**Supplementary Information:**

The online version contains supplementary material available at 10.1186/s12889-021-11549-3.

## Background

In 2019, the International Diabetes Federation (IDF) estimated that more than 8% of the global population aged 20–79 have diabetes, of which 90% have type 2 diabetes (T2D). In countries of the Middle East and North Africa (MENA) region, it is estimated that the number of people with diabetes will increase by 96% by 2045, which will be the second-highest regional increase after Africa (143%). The negative impact of diabetes on health care system expenditures, population productivity and quality of life is of great concern.

In Oman, a national survey in 2017 reported non-communicable (NCD)-related risk factor prevalence as obesity 66%, high blood pressure 32.2%, cholesterol ≥5 mmol/L 37%, insufficient physical activity 42%, and unhealthy diet 56% [1]. The prevalence of diabetes had shown a dramatic increase from 8.3 to 11.6% to 12.3% in 1991, 2000, and 2008, respectively and recent estimates are in the order of 15%, exceeding the global rates [2, 3]. In addition to that, two-thirds of adults with diabetes are overweight or obese.

Physical inactivity is estimated to be the principal cause for 27% of diabetes, and 30% of ischemic heart disease [4]. Similarly, greater sitting time known as sedentary behavior (SB) is considered an independent risk factor for diabetes, cardiovascular disease, and all-cause mortality [5–8]. Sitting more than 8 h/day leads to increase risk of all-cause mortality even among those achieving the recommended 150 min/week of moderate to vigorous physical activity (PA) [9].

Overweight or obesity is the fifth leading risk factor for global death and the third in high income countries [10]. Around 3.4 million adults die each year as a result of being overweight or obese [11, 12]. Obesity has been attributed to the burden of 44% of diabetes, 23% of the ischemic heart disease and up to 41% of certain cancers [13].

The evidence for the impact of lifestyle modifications including diet, physical activity (PA), and reducing sitting time in controlling blood glucose levels and in the overall management of diabetes is well documented especially within clinical setting [14–16]. Increasing PA, together with reducing and regularly interrupting sitting time are now considered cornerstones of diabetes management [17]. However, a recent study in Oman showed that only 21.6% of adults with T2D achieved the recommended levels of PA - defined as at least 150 min of moderate-intensity aerobic physical activity throughout the week- and the average sitting time was > 12 h/ day [18]. Additionally, more than half of adult Omanis consume unhealthy diet leading to excess weight.

The multi-center Look AHEAD project has provided a high quality evidence about the effectiveness of lifestyle modification in diabetes management [19, 20]. The primary objective of this randomized controlled trial was to examine the long-term effects of lifestyle interventions- defined as 175 min of PA per week and a calorie counting with inclusion of fat gram counting and portion controlled- on cardiovascular morbidity and mortality in 5145 overweight or obese participants with diabetes. Despite the debatable lack of effectiveness of the intensive lifestyle intervention program on the cardiovascular morbidity and mortality, significant positive effects on weight, waist circumference, physical fitness and HbA1C were highly noted [21–23].

Current evidence on lifestyle including PA and dietary interventions in diabetes care comes mostly from Western countries, however results were diverse [24]. There was a variation in the type of interventions in terms of setting (clinical vs community), intervention methods (PA consultations, exercise sessions, or use of technology) and duration (short of 3–6 months vs long ≥12 months). Successful interventions have demonstrated that in obese patients with diabetes, PA and more extreme dietary energy restriction with very low-calorie diets can reduce HbA1c to < 6.5% and fasting glucose to < 126 mg/dL without pharmacological therapy or surgical procedures [25, 26].

Incorporating behavior change techniques (BCTs) in PA interventions has been shown to effectively move individuals from ‘inactive’ to ‘active’ stages of change for PA [27] and in weight management [28]. Whilst there is a large number of BCT’s, these have been standardised by Abraham and Michie [29] to assist the development of lifestyle interventions [30]. Avery et al. (2015), distinguished five context-appropriate BCTs for use during time-constrained consultations in diabetes care. These include “prompt focus on past success”, “barrier identification”, “use of follow-up prompts”, “providing information on where and when to perform the behaviour” and “prompt review of behavioural goals of PA” [27].

Consistent with the socio-ecological models of health behaviour (PA and SB) [31–33], and the Behavioural Change Wheel (BCW) model [30], the work presented in this proposal is a continuation of series of published studies to specifically promote healthy lifestyle in adults with diabetes primary care providers in Oman [18, 34–36]. Results showed that PA consultations linked to BCTs, devices to support walking such as pedometers, and use of phone applications were significantly associated with positive changes in PA behaviour including sitting time. The current proposal, in addition to PA, integrates dietary advice and includes innovative use of technology to enhance excess weight reduction and glycemic control in adults with diabetes. This proposal also integrates SB which is a growing research area that is under reported in Arabic speaking countries namely Oman. Results from this study is hoped to influence changes in the current national diabetes management policies and guidelines by providing evidence on effective lifestyle interventions using artificial intelligence within routine diabetes primary health care settings and highlighting the capacities needed to do so.

### Hypothesis

Other effective methods are needed to promote healthy lifestyle among Omani patients with T2D. We expect that a multi component intervention including phone consultations, use of pedometers and a smart phone application are effective in behavior change to promoting PA and healthy diet among patient with T2D in primary care compared with usual care.

### Primary objective

To evaluate the effectiveness of phone consultations and use of pedometers and a smart phone application in changing to positive behavior of PA and healthy diet and their impact on reducing excess weight [measuring body mass index (BMI)] and glycemic control (HbA1c).

### Secondary objectives

Estimate the impact on cardio-metabolic risk factors by measuring blood pressure and lipid profile at baseline and 12 months.

Estimate the impact on developing diabetes complications, by performing blood tests for kidney function, screen for retinopathy, diabetic foot exam findings at baseline and 12 months.

Estimate the impact of the intervention on change in PA and sitting time at 12 months follow up from baseline.

Estimate the impact of the intervention on the course of diabetes drugs/treatment plans at 12 months.

To examine the feasibility of the intervention (content, delivery and satisfaction) to the participants and care providers.

## Methods

### Study setting

A one year 1:1 cluster randomised control trial in eight primary care facilities in A’Seeb Wilayat, which will be randomly allocated (using random numbers table generated in SPSS v21) to either the intervention components or usual care.

### Eligible criteria

#### Inclusion criteria

- Adults aged18–65 years of age
- Diagnosed with diabetes and overweight/ Obese
- Attending (registered) health facilities for at least 6 months
- HbA1c > 7% (≥ 48 mmol/mol) according to Oman diabetes management guidelines.
- Assessed by project officer as having inactive behavior
- No contraindication to physical activity
- Not on a restricted diet.
- Able to speak and read Arabic
- Willing and able to provide written informed consent to the study
- Can use phone applications

#### Exclusion criteria

Patients with:

- A history of myocardial infarction in the previous 6 months
- A serum creatinine > 140 mmol/L (from previous recorded readings in the electronic health information system)
- Diabetic foot ulcers or at high risk of ulcer (severe peripheral neuropathy)
- Repeated hypoglycaemia or severe hypoglycaemia in previous 12 months
- No internet access for the phone application
- Physical activity > 150 min per week
- HbA1c ≤7%

### Recruitment of project officers

At least three project officers will be recruited at each health facility from the existing health care providers (doctors/nurses/dieticians/health educators). Project officers will receive study specific training on the recruitment procedures, screening the participants, recording outcome measurements, and delivering the intervention to the patients.

All eligible patients in the selected health centers will be informed about the study by the project officers and invited to participate in the study. Interested patients will be screened for physical inactivity.

### Training of project officers

All project officers will be trained on study procedures and assessment tools. A Bespoke training on using pedometers and the app will be delivered by national experts in Oman (proposed dates March 2021). The training will target project officers who will deliver the consultations not those taking the study measurements.

### Blinding

Except for the socio-demographic data at baseline, all measures will be collected by nurses who will be blinded from the study objectives and group allocation. Owing to the nature of this study, the project officers may not be blinded completely from study objectives, however they will not be involved in data entry and/or analysis.

### Randomisation

The study is a 1 year 1:1 cluster randomized controlled trial of the lifestyle intervention versus usual care. A cluster randomisation design will be used to minimize between group contamination by having the two groups (intervention and comparison) from independent health centres. Group allocation will be generated using a random numbers table generated in SPSS v21done by an independent statistician at MOH. All health facilities (8) will be randomised to deliver either the intervention (*n* = 4) or usual care (n = 4). Health facilities will be informed of their allocation verbally by the project investigator and will receive an envelope containing invitation letter and project materials.

### Recruitment of patients

**Fig. 1:**
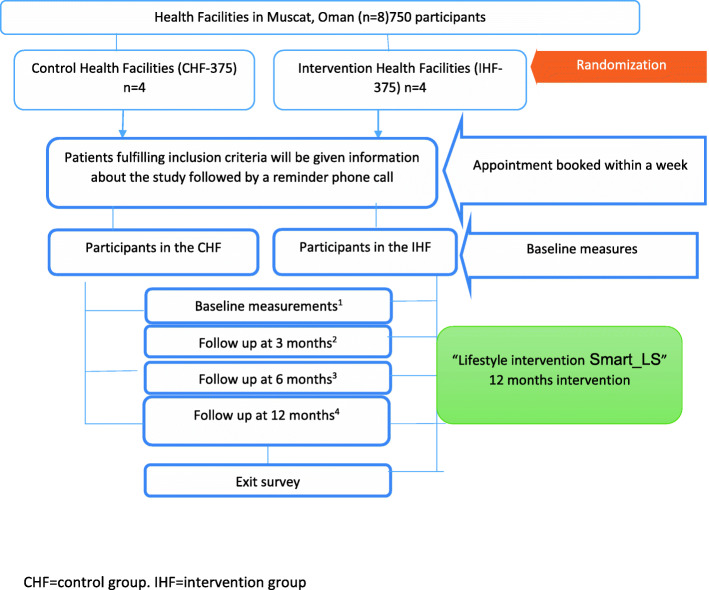
Patient flow chart. CHF = control group. IHF = intervention group

Recruitment will take place over a 2-month period. Inactive patients fulfilling the inclusion criteria will be provided with a participant information sheet about the study given by the project officers. Subsequently, an appointment will be given to all potential participants to attend a wellbeing clinic for baseline measures, linked to their diabetes clinic, within a week. A telephone call will be made to all willing participants to remind them of their appointment and ensure activation of the phone app. At the baseline visit the project officers will seek written informed consent and log any eligible individuals who decline participation (Fig. 1).

Recruitment will be monitored on monthly basis and efforts to reduce loss to follow-up will be made by sending encouraging messages regularly to participants. Participants not attending their appointment will be called to consider rescheduling appointments, otherwise will be considered as withdrawal cases.

### Intervention group

All participants will receive the smart app that contains personalised PA and diet platforms, pedometer to measure weekly step counts and interactive platforms for peer motivation after registering them for to get an access username and password. In addition to that, patients will receive face to face visits to measure their anthropometric and metabolic parameters. Table 1 shows the timeline for each intervention component within the app.

**Table 1:** Intervention components

Intervention visits	Weeks												
	0^**a**^	4	8	12^**a**^	16	20	24^**a**^	28	32	36^**a**^	40	44	48
**Phone physical activity and diet phone consultations**													
**Pedometer step counts**													
**Smart_LS Interactive phone App**													

^a^Point of intervention delivery

### Phone consultations

Recruited participants will be offered individual phone consultations (maximum 20–30 min) by the trained project officers on 4 occasions (0, 3, 6 and 12 months) on PA and healthy diet.

### Pedometer

Participants will be given a pedometer at their baseline visit. Instructions on how to use the pedometer, how to record their daily steps and how to set daily step goals will be discussed by the project officers. Participants will be asked to set individual goals and report their step count in the interactive phone application over the period of 12 months.

### Bespoke phone application for PA and healthy diet

Participants receiving the intervention will be asked to share a phone application in order to report their PA and diet behaviours step counts at baseline, 3, 6, 9 and 12 months. Participants will be encouraged to communicate with their respected project officers and their peers (optional). The application will be designed, and managed by experts from higher collage of technology in Oman.

### Usual care group

In the control arm, doctors will be allowed to continue the regular counselling and prescription for diet and physical activity according to current national guidelines and existing practices. In these conventional clinical consultations, healthcare is provided according to existing knowledge; counselling is given at the individual clinician’s decision. We will ensure a pragmatic comparator that reflect typical routine practice, which allows for useful comparison of the intervention to the existing care.

### Outcome measures/assessment instruments

Table 2 describes all relevant outcomes, tools used and time at measurement.

**Table 2:** Outcome measures for pre-specified variables

Primary Outcome	Tool	When to measure	Type of variable
**Body weight (Kg)**	**Calibrated scales**	**B, 3F, 6F, 12F**	**continuous**
^**a**^**HbA1c (%)**	**Blood test (fasting sample)**	**B, 3F, 6F, 12F**	**continuous**
**Secondary Outcomes**			
**Socio-demographic data**	**Questionnaire**	**B**	**Binary / numerical**
**Height (m)**	**Stadiometer**	**B**	**continuous**
**Pedometer**	**Reporting step counts**	**B, 3F, 6F, 12F**	**numerical**
**GPAQ-MET-mins/week**	**Questionnaire**	**B, 3F, 6F, 12F**	**continuous**
**Diet assessment tool**	**Questionnaire**	**B, 3F, 6F, 12F**	**continuous**
**Lipid profile (mmol/l)**	**Blood test (venous fasted sample)**	**B, 3F, 6F, 12F**	**continuous**
**Blood pressure (mmHg)**	**Sphygmomanometer**	**B, 3F, 6F, 12F**	**continuous**
**Exit survey**	**Questionnaire (participants and project officers)**	**12F**	**description**

(B = baseline; 3F = 3 month follow-up; 6F = 6 month follow up; 12F = 12 month follow-up)

^a^while blood collection for HbA1c at 12 month is mandatory, it is only done at baseline, 3 and 6 month if missing from the electronic health information system or recorded within more than 4 months prior to the measurement visits

#### Sociodemographic information

A multi-section questionnaire will be available in the app will be used to interviewing the participants and collect basic information including gender, willayat, age, marital status, education, income, and job.

#### Physiological (medical history) data

This will include duration of diabetes, BMI, blood pressure, lipid profile, and presence of any comorbidities defined as cardiovascular, hyperlipidemia, thyroid abnormalities, renal, eye, musculoskeletal, or any other recorded condition in the electronic health information system coinciding with diabetes. Drug (treatment) history: type and dose will also be recorded.

#### Primary outcome

##### Physical activity

The Global PA questionnaire (GPAQ) will be used to assess physical activity based on time period shown in Table 1.

##### Nutrition assessment

Medical nutrition therapy for diabetes mellitus (MNT for diabetes) survey will be and nutrition status will be described in scores.

##### Body mass index

Every patient will have their weight and height measured using standardized and calibrated scales available in health centers at 4 time points (Table 1). In addition to that, waist circumference will also be measured using a measuring tape. The measurements will be taken by trained project nurses.

##### Glycated hemoglobin level (HbA1c)

Every patient will have their HbA1c level measured. Blood sample will be drawn by the project nurse, using sterilized vacutainers and samples collect in purple topped tube. Standardized and calibrated laboratory machines will be used to measure HbA1c level.

### Data collection and management

To evaluate the primary and secondary outcomes, all relevant data will be collected by the assigned project officers for data collection. Data will be recorded as described earlier at specified time points after randomisation. Drug history will be obtained from electronic records where available.

### Sample size calculation

The study is powered to detect a change considered to be both meaningful and plausible on the primary outcome. A sample size of 392 (196 in each arm) will allow measuring a between group difference of 7% in BMI and 0.6%in HbA1c over 12 months from baseline, within a power of 80%, significance level of 5%, [19, 37] and a standardised difference of 0.394 (i.e. 0.394 of a standard deviation can be detected) under an Intra-cluster correlation coefficient of 0.01). With a drop-out rate of 20% and a recruitment rate of 70% the required sample size is 750 (375 in each arm). The equation below was used to calculate the sample size based on evidence from nearby countries, the UAE [38].

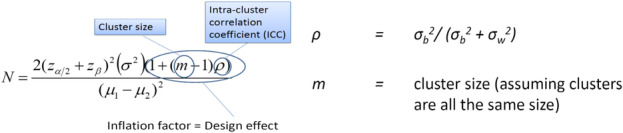



### Statistical analysis

The analysis plan was adopted for a previous RCT on physical activity in diabetes in Oman [36]. Once all participants have been randomised, descriptive statistics including t-test for continuous variables and chi square for binary variables will be used to compare the two group’s variables at baseline. Descriptive analysis will also be used for summary statistics on patient assessment, eligibility, recruitment, withdrawal.

The primary outcome which is the rate of PA and healthy diet score, BMI and glycated hemoglobin over 12 months for all participants in both trial arms at 3, 6, and 12 months will be measured, summarized and tabulated for both study arms. Graphical illustration (box plots, histograms and bar charts) will be used where appropriate and the distributions of all outcome measures will be checked and transformed where appropriate. The main analysis will be linear regression modeling to test the main hypothesis related to the superiority of the proposed intervention in promoting healthy lifestyle compared with control group. All outcomes will be analysed adopting an intention-to-treat principle and all persons will be analysed in the groups to which they were randomised. We will try to minimize missing of data collected by ensuring complete and accurate data entry, however multiple imputation on the longitudinal data will be performed if we face missing data. For secondary analyses, logistic regression will be used adjusting for clustering of patients within health care facilitates.

Analysis and reporting of the results will follow the Consolidated Standards of Reporting Trials guidelines (CONSORT) for reporting RCTs [39]. SPSS version 21 will be used for data analysis.

### Assessment of fidelity to protocol

- Monthly meetings will be conducted to discuss issues regarding the consultations, and measurements to ensure their compliance to intervention protocol.
- Attendance sheets will be reviewed and discussed.
- Crosschecking of 10% of phone PA consultation notes randomly selected by a recruited external assessor.

### Stakeholder involvement

At the beginning of the trial a meeting will be held with all Head of health facilities to discuss the practicalities of this intervention. Required approvals will also be sought from the director general of health services, Muscat. A monthly meeting and regular telephone messages with all the project officers will be conducted to facilitate smooth running of the project.

### Participant and public involvement

We will involve 2 to 3 patients with diabetes to share their perceptions and ideas about the practicality of using electronic applications in promoting healthy lifestyle. They are welcomed to join the study team meeting and discuss app components and outcome measures. The perspectives of caregivers will also be taken into consideration in testing and refining the study materials and procedures. At the end of the study, participants and care givers satisfaction with the intervention, including burden, will be assessed as part of the process evaluation.

Results of the main trial and other evaluation findings will be submitted for publication in international peer-reviewed journals and presented at international and regional scientific conferences and webinars.

Furthermore, the findings will be shared to the community through social media platforms to encourage community engagement in healthy lifestyle promotion.

## Discussion

Globally 72% of deaths are attributed to NCDs (4% due to diabetes vs 8% in Oman). Governments including Oman have endorsed nine global voluntary targets to reduce premature death from the four major NCDs (cardiovascular, diabetes, respiratory and cancer) by 25% by 2025 [40]. This project is targeted towards facilitating the global aims of: A) 25% relative reduction in the overall mortality from cardiovascular diseases, cancer, diabetes, or chronic respiratory diseases, B) 10% relative reduction in prevalence of insufficient physical activity and C) 80% availability of the affordable basic technologies and essential medicines required to treat major NCDs.

Oman has been chosen as one of the 12 countries to be monitored for combating NCDs and their risk factors including UN declaration 2011 for NCDs. In response, several strategic actions were put in place:

1. Launching the national policy for prevention and control of NCDs
2. Oman health vision 2050 including NCDs
3. Ministry of health policies and national five health development plans
4. The Oman national policy for diet and physical activity and health
5. The national NCDs screening program

To the best of our knowledge, this is the first study in Oman exploring the implementation/integration of lifestyle intervention methods within routine diabetes health care facilities. This proposal (longitudinal cluster randomised trial) meets the aim of the strategic research program for NCDs through providing:

1. Data on levels of physical behaviour including sedentary time in adults with diabetes.
2. Building partnership with other educational, governmental and organisations to promote healthy lifestyle.
3. Evidence on ways for implementation, up scaling and roll out of the intervention to other regions in Oman.
4. Opportunities for updating the current diabetes management policies and guidelines to meet the actual clinical needs.

## Conclusion

This research will help to inform current policies and practices of the Omani Ministry of Health for the use of a culturally acceptable healthy lifestyle promotional interventions as an integral part of care for clients with T2D within the primary health care setting using artificial intelligence.

## Supplementary Information

**Additional file 1.**

## Data Availability

Data generated from this study is not publicly available as this requires approval from the central research committee in Oman Ministry of Health, however it is available from the principal investigator on reasonable request.

## Abbreviations

T2D: Type 2 diabetes
PA: Physical activity
IDF: International Diabetes Federation
MENA: Middle East and North Africa
SB: Sedentary behavior
NCD: Non-communicable
BCTs: Behavior change techniques
BMI: Body mass index
