# Supplementary material for: Study protocol: behaviour change intervention to promote healthy diet and physical activity in overweight/obese adults with diabetes attending health care facilities in Muscat: a cluster rendomised control trial
Source: BMC Public Health. 2021 Aug 10;21:1529. doi: 10.1186/s12889-021-11549-3 (PMC8353738; doi:10.1186/s12889-021-11549-3)
Supplement: Supplementary file 1 — Additional file 1. [file 12889_2021_11549_MOESM1_ESM.pdf]

Physical activity data from the WHO (GPAQ):

Next I am going to ask you about the time you spend doing different types of physical activity in a typical week. Please answer these questions even if you do not consider yourself to be a physically active person. Think first about the time you spend doing work (paid or unpaid) e.g. study/training, household chores, harvesting food/crops, fishing or hunting for food, seeking employment.

In answering the following questions:

- **'Vigorous-intensity' activities require hard physical effort and cause large increases in breathing or heart rate.**
- **'Moderate-intensity' activities require some physical effort and cause noticeable increases in breathing or heart rate.**

Questions		Response	
**Work activity (excluding travel to and from work)**			
1.	Does your work involve vigorous-intensity activity that causes large increases in breathing or heart rate *[like carrying or lifting heavy loads, digging or construction work*] for at least 10 minutes continuously?	1. Yes<br>2. No	***If No, go to Q 4***
2.	In a typical week, on how many days do you do vigorous-intensity activities as part of your work?	Number of days	└─┘
3.	How much time do you spend doing vigorous-intensity activities at work on a typical day?	Hours : minutes	└─┴─┘ : └─┴─┘<br>hrs mins
4.	Does your work involve moderate-intensity activity that causes noticeable increases in breathing or heart rate such as brisk walking *[or carrying light loads*] for at least 10 minutes continuously?	1. Yes<br>2. No	***If No, go to Q 8***
5.	In a typical week, on how many days do you do moderate-intensity activities as part of your work?	Number of days	└─┘
6.	How much time do you spend doing moderate-intensity activities at work on a typical day?	Hours : minutes	└─┴─┘ : └─┴─┘<br>hrs mins
**7.**	**How much time do you spend at work/ working in a regular day?**	**Hours : minutes**	└─┴─┘ **:** └─┴─┘<br>**hrs mins**
**Travel to and from places (including to and from work)**			
The next questions exclude the physical activities at work that you have already mentioned. Now I would like to ask you about the usual way you travel to and from places. For example to work, for shopping, to market, to place of worship.			
8.		**1. Yes**	

	**Do you walk or use a bicycle (*pedal cycle*) for at least 10 minutes continuously to get to and from places?**	**2. No**	***If No, go to Q 11***
9.	In a typical week, on how many days do you walk or bicycle for at least 10 minutes continuously to get to and from places?	Number of days	└─┘
10.	How much time do you spend walking or bicycling for travel on a typical day?	Hours : minutes	└─┴─┘ : └─┴─┘ hrs mins

Recreational activities

The next questions exclude the work and transport activities that you have already mentioned. Now I would like to ask you about sports, fitness and recreational activities (leisure).

11.	Do you do any vigorous-intensity sports, fitness or recreational (*leisure*) activities that cause large increases in breathing or heart rate [*like running or football]* for at least 10 minutes continuously?	1.Yes<br>2.No	***If No, go to Q 14***
12.	In a typical week, on how many days do you do vigorous-intensity sports, fitness or recreational (*leisure*) activities?	Number of days	└─┘
13.	How much time do you spend doing vigorous-intensity sports, fitness or recreational activities on a typical day?	Hours : minutes	└─┴─┘ : └─┴─┘ hrs mins
14.	Do you do any moderate-intensity sports, fitness or recreational (*leisure*) activities that cause noticeable increases in breathing or heart rate for at least 10 minutes continuously?	1. Yes<br>2. No	***If No, go to Q 17***
15.	In a typical week, on how many days do you do moderate-intensity sports, fitness or recreational (*leisure*) activities?	Number of days	└─┘
16.	How much time do you spend doing moderate intensity sports, fitness or recreational activities on a typical day?	Hours : minutes	└─┴─┘ : └─┴─┘ hrs mins

Sitting time

The following question is about sitting or reclining at work, at home, getting to and from places, or with friends including time spent [sitting at a desk, sitting with friends, travelling in car, bus, train, reading, playing cards or watching television], but do not include time spent sleeping.

17.	How much time do you usually spend sitting or reclining on a typical day?	Hours : minutes	└─┴─┘ : └─┴─┘ hrs mins

بيانات النشاط البدني من منظمة الصحة العالمية (GPAQ):

بعد ذلك سأقوم بتوجيه أسئلة لك عن الوقت الذي تقضيه في ممارسة مختلف أنواع النشاط البدني خلال أسبوع اعتيادي، يرجى الإجابة على الأسئلة حتى وإن كنت لا تعتبر نفسك شخصا نشطا بدنيا، فكر أولا في الوقت الذي تصرفه في العمل (سواء كان مدفوع الأجر أم لا) مثل الدراسة أو التدريب والأعمال المنزلية والبستنة والصيد أو البحث عن وظيفة.

وعند الإجابة على الأسئلة:

- فإن الفعاليات التي تتطلب نشاطا شديدا هي التي تتطلب جهدا بدنيا كبيرا وتتسبب في ارتفاع كبير في معدلات التنفس ونبضات القلب.
- الفعاليات التي تتطلب نشاطا معتدلا هي التي تتطلب جهدا بدنيا إلى حد ما وتتسبب في ارتفاع ملحوظ بالتنفس ونبضات القلب.

	الأسئلة	الاستجابات		
	**نشاط العمل (باستثناء السفر من وإلى العمل)**			
١.	هل يشمل عملك ممارسة نشاط بدني شديد بما يتسبب في ارتفاع كبير بمعدل التنفس ونبضات القلب [مثل حمل أشياء ثقيلة أو الحفر أو أعمال البناء] لفترة ١٠ دقائق متصلة على الأقل؟	١- نعم<br>٢- لا		**إن كانت الإجابة لا فانتقل إلى السؤال رقم ٤**
٢.	في خلال أسبوع اعتيادي، كم مرة تمارس نشاطا بدنيا شديدا كجزء من عملك؟	عدد الأيام	└──┘	
٣.	كم الوقت الذي تقضيه وأنت تمارس النشاط البدني الشديد في العمل خلال يوم واحد	الدقائق: الساعات	└──┴──┘ : └──┴──┘<br>دقائق ساعات	
٤.	هل يشمل عملك ممارسة نشاط بدني معتدل بما يتسبب بارتفاع ملحوظ في معدل التنفس ونبضات القلب مثل المشي السريع [أو حمل أشياء خفيفة] لفترة ١٠ دقائق متصلة على الأقل؟	١- نعم<br>٢- لا		**إن كانت الإجابة لا فانتقل إلى السؤال ٨**
٥.	في خلال أسبوع اعتيادي، كم مرة تمارس نشاطا بدنيا معتدلا كجزء من عملك؟	عدد الأيام	└──┘	
٦.	كم الوقت الذي تقضيه وأنت تمارس النشاط البدني المعتدل في العمل خلال يوم واحد	الدقائق: الساعات	└──┴──┘ : └──┴──┘<br>دقائق ساعات	
٧.	كم من الوقت تقضيه في العمل/وأنت تعمل في يوم اعتيادي واحد	الدقائق: الساعات	└──┴──┘ : └──┴──┘<br>دقائق ساعات	
	**التنقل من وإلى مختلف الأماكن (يشمل التنقل من وإلى العمل)**			
	الأسئلة التالية تستثني النشاط البدني المذكور سابقا في موقع العمل، الآن أريد أن أسألك عن الطريقة المعتادة للتنقل من وإلى الأماكن المختلفة مثل العمل والتسوق وشراء الحاجيات ودور العبادة.			
٨.	**هل تمشي أو تستعمل الدراجة الهوائية لفترة ١٠ دقائق متواصلة على الأقل للوصول إلى حيث تريد؟**	**١- نعم**<br>**٢- لا**		**إن كانت الإجابة لا فانتقل إلى السؤال ١١**

.٩	خلال الأسبوع الاعتيادي، في كم يوم تمشي أو تستخدم الدراجة الهوائية لفترة ١٠ دقائق متواصلة على الأقل؟	عدد الأيام |__|
.١٠	كم من الوقت تقضيه وأنت تمشي أو تستعمل الدراجة الهوائية للتنقل خلال اليوم الاعتيادي؟	الدقائق: الساعات |__|__| : |__|__| دقائق ساعات

الأنشطة الترفيهية

الأسئلة التالية تستثني ما ذكر في أعلاه عن العمل والتنقل من مكان لآخر، الآن أريد أن أسألك عن الرياضات والأنشطة التي تمارسها لتحافظ على رشاقتك ولتستمتع بوقتك.

.١١	هل تمارس أي رياضة أو نشاط ترفيهي يتطلب بذل مجهود بدني شديد ويتسبب في ارتفاع كبير في معدل التنفس ونبضات القلب مثل الركض وكرة القدم لفترة ١٠ دقائق متصلة على الأقل؟	١- نعم<br>٢- لا	**إن كان الجواب لا فانتقل إلى السؤال ١٤**
.١٢	خلال الأسبوع الاعتيادي، في كم يوم تمارس أي رياضة أو نشاط ترفيهي يتطلب بذل مجهود بدني شديد ؟	عدد الأيام	|__|
.١٣	كم من الوقت تقضيه في ممارسة رياضة أو نشاط ترفيهي يتطلب بذل مجهود بدني شديد خلال اليوم الاعتيادي؟	الدقائق: الساعات	|__|__| : |__|__| دقائق ساعات
.١٤	هل تمارس أي رياضة أو نشاط ترفيهي يتطلب بذل مجهود بدني معتدل ويتسبب في ارتفاع ملحوظ في معدل التنفس ونبضات القلب القدم لفترة ١٠ دقائق متصلة على الأقل؟	٣- نعم<br>٤- لا	**إن كان الجواب لا فانتقل إلى السؤال ١٧**
.١٥	خلال الأسبوع الاعتيادي، في كم يوم تمارس أي رياضة أو نشاط ترفيهي يتطلب بذل مجهود بدني معتدل ؟	عدد الأيام	|__|
.١٦	كم من الوقت تقضيه في ممارسة رياضة أو نشاط ترفيهي يتطلب بذل مجهود بدني معتدل خلال اليوم الاعتيادي؟	الدقائق: الساعات	|__|__| : |__|__| دقائق ساعات

وقت الجلوس

الأسئلة التالية هي عن الجلوس أو التمدد في العمل والمنزل وأثناء التنقلات ومع الأصدقاء ويشمل الأوقات التي تقضيها جالسا على المكتب ومع الأصدقاء وأثناء الجلوس بالسيارة أو الباص أو القطار أو أثناء القراءة ولعب الورق ومشاهدة التلفاز ولكن لا يشمل الوقت المصروف بالنوم

.١٧	كم من الوقت تصرفه عادة أثناء الجلوس أو التمدد في خلال يوم اعتيادي واحد؟	الدقائق: الساعات	|__|__| : |__|__| دقائق ساعات
